# A New Ethylene-Responsive Factor *CaPTI1* Gene of Pepper (*Capsicum annuum* L.) Involved in the Regulation of Defense Response to *Phytophthora capsici*

**DOI:** 10.3389/fpls.2015.01217

**Published:** 2016-01-08

**Authors:** Jing-Hao Jin, Huai-Xia Zhang, Jun-Yi Tan, Ming-Jia Yan, Da-Wei Li, Abid Khan, Zhen-Hui Gong

**Affiliations:** College of Horticulture, Northwest A&F UniversityYangling, China

**Keywords:** pepper, ethylene-responsive factor (ERF), *Phytophtora capsici*, relative expression, virus-induce gene silencing (VIGS)

## Abstract

Ethylene-responsive factors (*ERF*) are usually considered to play diverse roles in plant response to biotic and abiotic stresses. In this study, an *ERF* gene *CaPTI1* was isolated from pepper transcriptome database. *CaPTI1* contains an open reading frame (ORF) of 543 bp, which encodes a putative polypeptide of 180 amino acids with a theoretical molecular weight of 20.30 kDa. Results of expression profile showed that *CaPTI1* had a highest expression level in roots and this gene could not only response to the infection of *Phytophthora capsici* and the stresses of cold and drought, but also be induced by the signaling molecule (salicylic acid, Methyl Jasmonate, Ethephon, and hydogen peroxide). Furthermore, virus-induce gene silencing (VIGS) of *CaPTI1* in pepper weakened the defense response significantly by reducing the expression of defense related genes *CaPR1*, *CaDEF1* and *CaSAR82* and also the root activity. These results suggested that *CaPTI1* is involved in the regulation of defense response to *P. capsici* in pepper.

## Introduction

Pepper (*Capsicum annuum* L.) is an agriculturally important vegetable crop worldwide. While the production of pepper is severely threatened by a variety of diseases, especially the soil borne disease *Phytophthora* blight which is caused by *Phytophthora capsici* (Hausbeck and Lamour, 2004; Granke et al., 2012). *P. capsici* could not only attack pepper plants, but also other crops such as cucurbits, tomato, eggplant, snap and lima beans (Lamour et al., 2012). *P. capsici* could affect the plant at any stage of development and cause damping-off, seedling blight, foliar blight and wilting follow by plant death. Infection on mature plants materializes as dark, rapidly expanding, water-soaked lesions (Baysal et al., 2005). Chemical and biological control are the most common strategies used to prevent the spreading of *P. capsici* (Oelke et al., 2003). Several fungicides have been reported to be effective against *P. capsici* on vegetable crops (Matheron and Porchas, 2000; Babadoost and Islam, 2003; Kousik et al., 2011). Although many studies have revealed that resistance to *P. capsici* is polygenic and could be controlled by quantitative trait loci (QTL) (Thabuis et al., 2003; Kim et al., 2008; Rehrig et al., 2014), little is known about pepper–*P. capsici* interactions at the molecular and genetic levels.

Plants have evolved many sophisticated defense mechanisms by activating multiple defense pathways in order to protect themselves from pathogen infection and cope with the pathogen invasion. salicylic acid (SA), Jasmonate (JA), and Ethylene (ET) are frequently induced in response to infection with various pathogens. The balance of these hormones is dependent on the recognized pathogen and plays a vital role in fine tuning appropriate defense responses (Dong, 1998; Dangl and Jones, 2001). SA typically mediates basal defense to biotrophic pathogens and is essential for the rapid activation of local and systemic resistance (Loake and Grant, 2007; Vlot et al., 2009). While JA controls defense reactions to necrotrophs. Together with ET, JA generally regulates induced systemic resistance (ISR) (Glazebrook, 2005; Alazem and Lin, 2015). ET may regulate pathogenesis-related (PR) genes expression through *ERFs*, and *ERFs* were supposed to act as either transcriptional activators or repressors of GCC-box mediated gene expression and be involved in the regulation of plant defense mechanisms (Fujimoto et al., 2000; Chakravarthy et al., 2003; Gutterson and Reuber, 2004; Yang et al., 2005; Alazem and Lin, 2015).

*ERFs* were firstly isolated in tobacco and found to regulate the expression of *PR* genes through binding specifically to the GCC-box (Ohmetakagi and Shinshi, 1995). Then the *ERFs* in other plants were constantly identified, such as *AtERFs*, *StERFs* and *LeERFs* (Zhou et al., 1997; Fujimoto et al., 2000; Wang et al., 2015). Fujimoto et al. (2000) isolated five *AtERFs* which respond to extracellular signals and modulate GCC box-mediated gene expression. Then Brown et al. (2003) found that *AtERF2* possibly play important role in regulating JA-dependent defense response via interaction with the GCC-box. The *ERFs* in tomato, *Pti4*, *Pti5*, and *Pti6* which were interacted with the resistance (R) gene *Pto* kinase were identified by yeast two-hybrid assays (Zhou et al., 1997). Further research found these *ERFs* could induced by *Pseudomonas* bacterium and also specifically recognize and bind to GCC-box of *PR* proteins (Thara et al., 1999). Moreover, *Pti4*, *Pti5*, and *Pti6* were suggested to indirectly regulate the SA response through interaction with other *TFs*, and activate the expression of *PR* genes (Gu et al., 2002; Chakravarthy et al., 2003). The over-expression of *Pti5* in tomato demonstrated the positive role of *Pti5* in defense genes regulation and disease resistance (He et al., 2001). Recently research revealed that *Pti5* contributes to potato aphid resistance in tomato independent of ethylene signaling (Wu et al., 2015). Besides, *NtTsi1* and *CaERFLP1* were also isolated and demonstrated to bind to the GCC-box (Park et al., 2001; Lee et al., 2004). Over-expression of *NtTsi1* in transgenic hot pepper plants induced constitutive expression of several *PR* genes in the absence of stress or pathogen treatment and enhanced the disease resistance to *Xanthomonas campestris pv. vesicatoria* and also the *P. capsici* (Shin et al., 2002).

Most of researches suggest that *ERFs* were involved in disease resistance responses as activators. There were also some *ERFs* whose protein contained an ERF-associated amphiphilic repression (EAR) motif in the C-terminal as repressors, such as *NtERF3*, *AtERF3*, *AtERF4* and *StERF3* (Ohta et al., 2001; McGrath et al., 2005; Tian et al., 2015). But recently Dong et al. (2015) found that the *GmERF5* which has an EAR motif could not only significantly induced by *P. sojae*, ETH, ABA and SA, but also bind to the GCC-box. Besides, transgenic soybean of *GmERF5* exhibited significant enhanced resistance to *P. sojae* and positively regulated the expression of the *PR* genes.

Although much progress has been made in elucidating the functional properties of *ERFs*, there was less research about *ERFs* in pepper. Previously, the *ERFs CaCBF1A*, *CaCBF1B* and *CaDREBLP1* who mainly response to environmental stresses (low temperature and drought) were isolated (Kim et al., 2004; Hong and Kim, 2005). In this study, an *ERF* gene, *CaPTI1* which was induced by pathogen was isolated from the transcriptome of interactions between pepper and *P. capsici*. The sequence character and structure were analyzed firstly. Then the expression profile of *CaPTI1* was analyzed by quantitative Real-Time-PCR (qRT-PCR). Furthermore, in past most of the virus-induced gene silencing (VIGS) concentrated in leaves but here we have performed in leaves as well as in roots of pepper to investigate the function of *CaPTI1* gene.

## Materials and Methods

### Plant Materials and Growth Conditions

Pepper cultivar Y3 was used in this study, which was provided by pepper breeding group in Northwest A&F University, China. First presoaking of the seeds was done and when 80% of seeds germinated, they were transferred to plastic pots having growing media [grass charcoal/perlite (3/1, v/v)]. Pepper plants were grown in a growth chamber with a 16 h light and 8 h dark photoperiod at 25°C.

### Pathogen Preparation and Inoculation

The virulent (HX-9) and avirulent strains (PC) of *P. capsici*, which was compatible and incompatible to cultivar Y3 respectively, were used in this research. Both of the strains were grown on potato dextrose agar (PDA) medium in the dark at 28°C for 7 days as described previously by Zhang et al. (2013). Then zoospores of *P. capsici* were induced by chilling at 4°C for 30 min and then incubated at room temperature for 30–60 min. The zoospores were collected by filtering through four layers of cheesecloth. After the zoospores were collected, a haemocytometer was used to adjust the concentration to 1 × 10^5^ zoospores mL^-1^ with sterile water.

For the VIGS of *CaPTI1* gene, pepper plants were inoculated with 3 mL zoospore suspension of *P. capsici* virulent strain HX-9 (1 × 10^5^ zoospores mL^-1^) using root drenching method. While for inoculation of detached leaves, third to fifth leaf from top of the control (*pTRV2:00*) and *CaPTI1* silenced plants were taken and sterilized with 75% ethanol for 1 min, washed three times with sterile water, then 20 μL zoospore suspension (1 × 10^5^ zoospores mL^-1^) of HX-9 were injected with a needleless syringe. After inoculation the leaves were kept in petri dishes and sealed with parafilm immediately. All the plants inoculated with *P. capsici* were put in a growth chamber at 28°C with a photoperiod of 16 h light/8 h dark and the relative humidity was kept 60%.

### Cloning and Sequence Analysis of *CaPTI1* Gene

Total RNA was extracted from the roots of Y3 plants which were inoculated with virulent strain of *P. capsici* and reverse transcribed into cDNA using a PrimeScript^TM^ Kit (Takara, Bio Inc, China) following the manufacturer’s protocols, and then the cDNA was used as a template to isolate the *CaPTI1* gene according to the transcriptome database of compatible and incompatible interactions of pepper (Y3) with *P. capsici*. After that *CaPTI1* gene was cloned into pMD19-T vector (Takara) and sequenced by Sangon Biotech (Shanghai) Co. Ltd.

The molecular weight (MW) and isoelectric point (pI) of *CaPTI1* were predicted by ExPASy^1^. Conserved domain of *CaPTI1* protein was identified in Conserved Domain of NCBI^2^. The full length of *CaPTI1* and other *ERF* proteins were used to construct the neighbor joining phylogenetic tree by MEGA 6.05 with 1000 bootstrap replicates. Multiple sequence alignments of the AP2/ERF domain in *CaPTI1* and other *ERFs* were performed by DNAMAN 5.0.

### *CaPTI1* Gene Expression Profile Analysis

To evaluate the expression levels of *CaPTI1* gene in different organs, the roots, stems, leaves, flowers, and immature fruits were collected from Y3 pepper plants, then frozen in liquid nitrogen and kept at -80°C for further RNA extraction and gene expression analysis.

Pepper plants at the six-true leaf stage were used for *P. capsici* inoculation and treatments of plant signaling molecule (ETH, SA, MeJA, and H_2_O_2_) and environmental stresses (drought and cold). For *P. capsici* inoculation treatment the root-drenching method was used as described previously (Zhang et al., 2013). Then leaves and roots from infected plants were collected at 0, 6, 12, 24, 48, 72, and 96 h after inoculation. All samples were collected and analyzed with three biological replicates.

In case of plant signaling molecule treatments, pepper plants were sprayed with 10 mM ETH, 5 mM SA, 50 μM MeJA or 10 mM H_2_O_2_ solutions, while in case of drought stress treatment, the roots of seedlings were watered with 0.4 M mannitol solutions. Pepper plants were placed at 4°C for cold stress treatment. Control plants were sprayed with sterile water. Pepper plants in above treatments were placed in a chamber at 25°C (except cold stress) under a 16 h light/8 h dark photoperiod cycle with 60% relative humidity. Leaves from treated plants were collected at 0, 3, 6, 9, 12, and 24 h after treatment, and frozen in liquid nitrogen immediately and kept at -80°C for RNA extraction. All samples were collected and analyzed with three biological replicates.

### RNA Isolation and Quantitative RT-PCR for Gene Expression Analysis

Total RNA was extracted from collected sample after various stress treatments using Trizol (Invitrogen) method. Then the first strand cDNA was synthesized using of prime script TM kit (Takara) following the manufacturer’s protocols. The NanoDrop instrument (Thermo Scientific NanoDrop 2000C, USA) was used to measure the concentration of cDNA. Later on qRT-PCR was carried out using SYBR^®^ Premix Ex Taq^TM^ II (TaKaRa) in an iCycler iQ^TM^ Multicolor PCR Detection System (Bio-Rad, USA). The amplification cycling parameters of qRT-PCR were as follow: 95°C for 1 min and followed by 40 cycles at 95°C for 10 s, 56°C for 15 s, and 72°C for 15 s. All the primers used for qRT-PCR were shown in **Supplementary Table S1**. Relative expression of genes was calculated as described by Livak and Schmittgen (2001) and *CaUbi3* gene was used as the reference gene in this study (Wan et al., 2011).

### VIGS of *CaPTI1* in Pepper

For VIGS assay, the 3′-untranslated region (UTR) of *CaPTI1* gene was selected to construct the combined vector *pTRV2:CaPTI1* and then this combine vector was used for subsequent VIGS experiment to ensure the specific silencing of *CaPTI1* (primers used for vector construction were shown in **Supplementary Table S1**). Then the *pTRV1*, *pTRV2* (negative control), *pTRV2:CaPDS* (positive control) (Wang et al., 2013a) and also the combined vector *pTRV2:CaPTI1* was transformed into *Agrobacterium tumefaciens* strain GV3101 by the freeze-thaw method. Agrodrench and leaf infiltration methods were used to silence *CaPTI1* in pepper (Ryu et al., 2006; Velasquez et al., 2009). The growth condition used for pepper seedling after inoculation was as described by Wang et al. (2013a). After inoculation for about 5 weeks, leaves and roots from *CaPTI1* silenced and control plants were collected for the analysis of silencing efficiency and expression level of relative defense genes. All experiments were performed and analyzed with three biological replicates.

### Determination of Root Activity

The root activity was measured by a modified triphenyltetrazolium chloride (TTC) method (Ou et al., 2011). Before the TTC tests, the roots of silenced and control plants at different time points after inoculation with virulent strain of *P. capsici* were collected and washed with sterile water. Then the root surfaces were dried carefully with absorbent papers. After that root activity was measured by a modified TTC method as described by Wang et al. (2013b). The root samples were placed in small beaker and incubated with 10 ml 1:1 (v/v) mixture of 1% TTC solution and 0.l M phosphate buffer (pH 7.0) at 37°C for 1 h in the dark. After the incubation, 2 ml of 1 M sulphuric acid was to added to inhibit the reaction. The root samples were rinsed twice with distilled water and ground in mortar with 3 ml ethyl acetate, in which the extracts of TTF (a derivative of TTC from the reduced reaction) were obtained and transferred to a 10 ml volumetric flask. The residues were rinsed with ethyl acetate and mixed with the earlier extracts, then the final volume was adjusted to10 ml. The absorption of the extraction was measured with a spectrophotometer at 485 nm. The control was similarly performed except the sulphuric acid was added first, the root sample second, and the mixture last. The reduced TTC amount was obtained from the standard curve and its intensity in the roots was calculated as follows: TTC reduction intensity [mg g^-1^ h^-1^] = reduced TTC amount/FW h (FW-fresh root mass; h-the incubation time). Each treatment was conducted in three independent experiments and each measurement was repeated three times.

### Statistical Analysis

All data were expressed as mean ± standard deviation (SD) of three independent replicates. Statistical Analysis System software (SAS 8.2, North Carolina State University, USA) was used for least significant difference (LSD) analysis and values of *p* < 0.05 was considered statistically significant. All experiments were performed and analyzed separately with three biological replicates.

## Results

### Cloning and Sequence Analysis of *CaPTI1*

**FIGURE 1:**
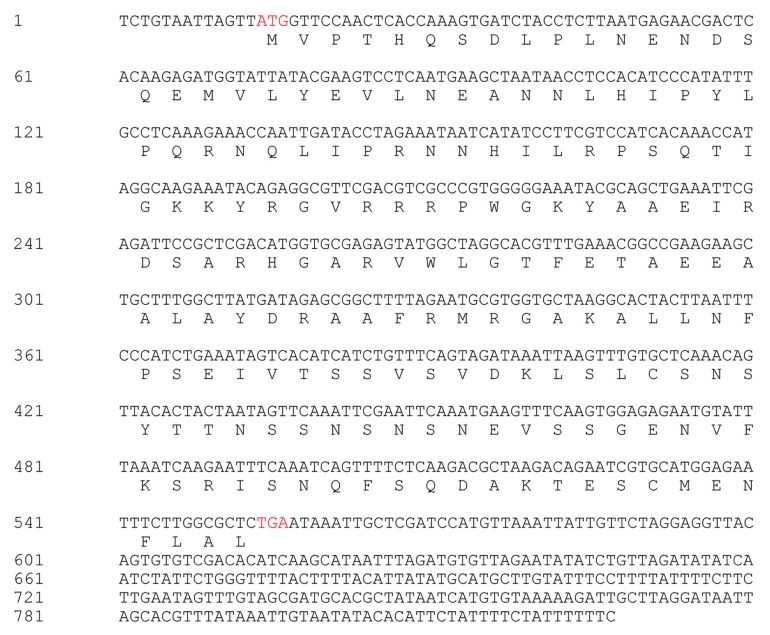
**The nucleotide sequence and deduced amino acid sequence of *CaPTI1***.

The *CaPTI1* gene (**Figure 1**) (GenBank No. KJ690096), which included an open reading frame (ORF) of 543 base pair (bp), was identified from pepper transcriptome database. The ORF of *CaPTI1* was predicted to encode a protein of 180 amino acids. The predicted molecular weight of the encoded protein was 20.30 kDa and *p*I 8.58. Sequence analysis found a single AP2/ERF domain in the *CaPTI1* protein.

**FIGURE 2:**
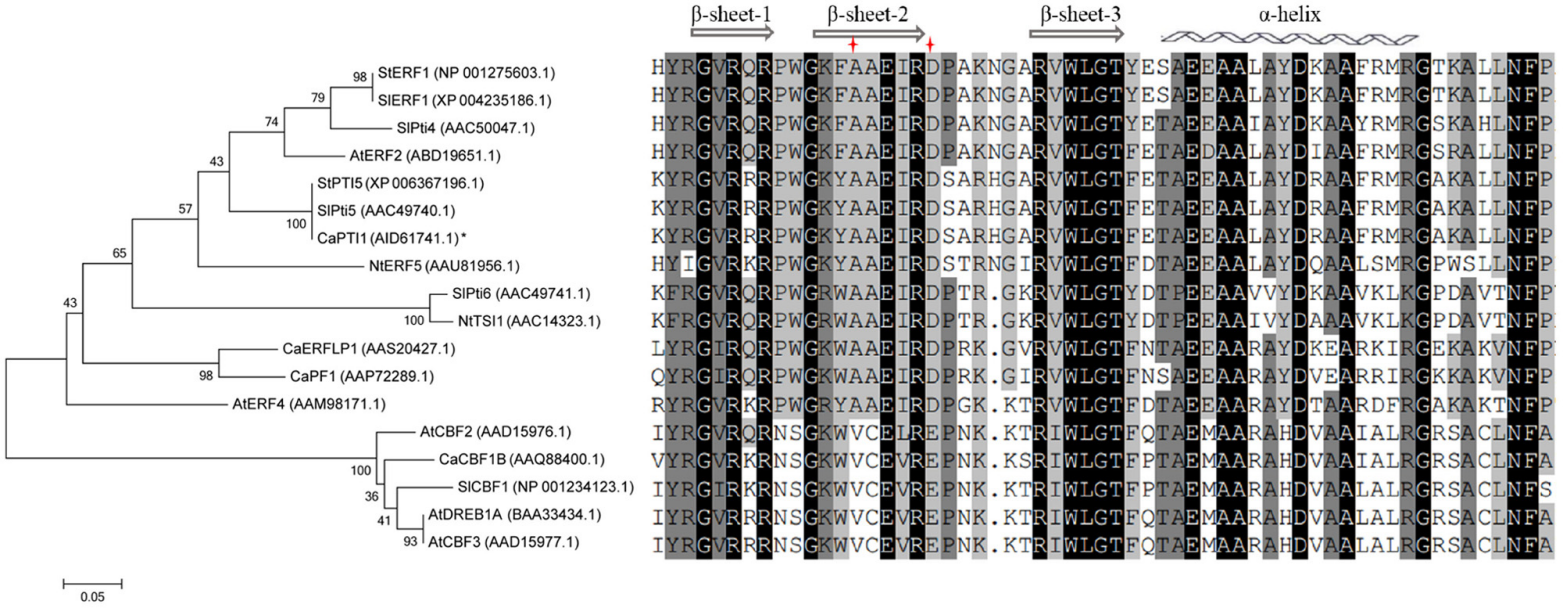
**Phylogenetic tree and multiple alignment of *CaPTI1* with other *ERFs***. Phylogenetic tree of *CaPTI1* with other Ethylene-responsive factors (ERF) proteins was generated by the MEGA 6.05 program with 1000 bootstrap replicates. GenBank No. of *ERFs* is in parentheses after each gene name. While the multiple alignment was conducted by DNAMAN5.0.

Furthermore the evolutionary relationships and multiple sequence alignment of *CaPTI1* in comparison with *ERF* proteins of other plants were also investigated (**Figure 2**). The result showed that *CaPTI1* had a more closely relationship with *SlPti5*. Multiple sequence alignment showed the conservation of AP2/ERF domain in *ERFs* from different plants. There were three β-sheets and an α-helix in the AP2/ERF domain (Allen et al., 1998; Djemal and Khoudi, 2015). The amino acids difference at position 14th and 19th in AP2/ERF domain was also existed in these *ERFs* (Sakuma et al., 2002).

### Tissue-Specific Expression of *CaPTI1* Gene in Pepper Plants

**FIGURE 3:**
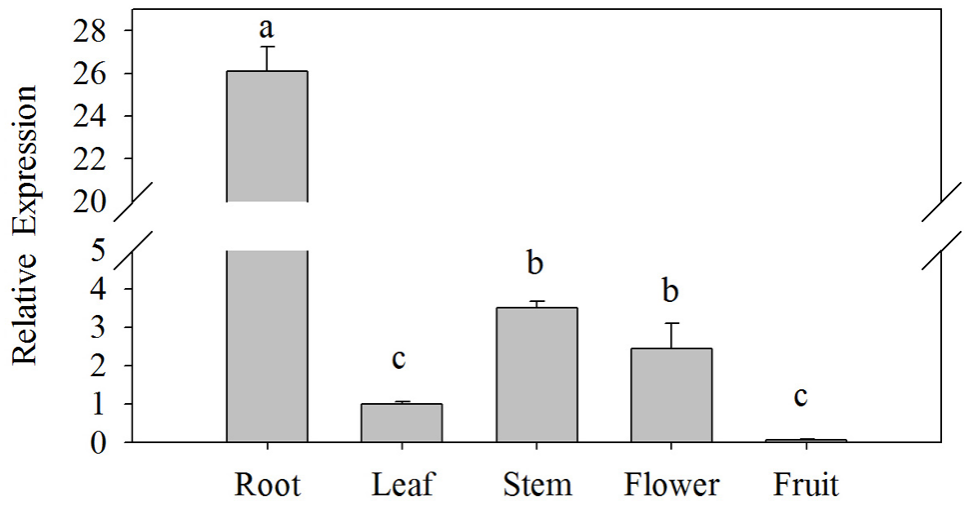
**Tissue specific expression of *CaPTI1* in pepper.** Error bars represent standard deviation (SD) for three independent replicates. Bars with different lower case letters indicate significant differences using Duncan’s multiple range test (*p* < 0.05).

The expression levels of *CaPTI1* gene in different tissues of Y3 plants were investigated by qRT-PCR. As shown in **Figure 3**, significant difference of *CaPTI1* gene expression was observed in different tissues. The highest expression was detected in roots, which was almost 26-fold of that in leaves. The expression level of *CaPTI1* gene in stems and flowers were also higher than that in leaves, which was 3.50 and 2.45-fold of that in leaves respectively. While lower expression was detected in fruits as compared to leaves which was statistically non-significant.

### Expression Analysis of the *CaPTI1* Gene in Response to *Phytophthora capsici* Infection

**FIGURE 4:**
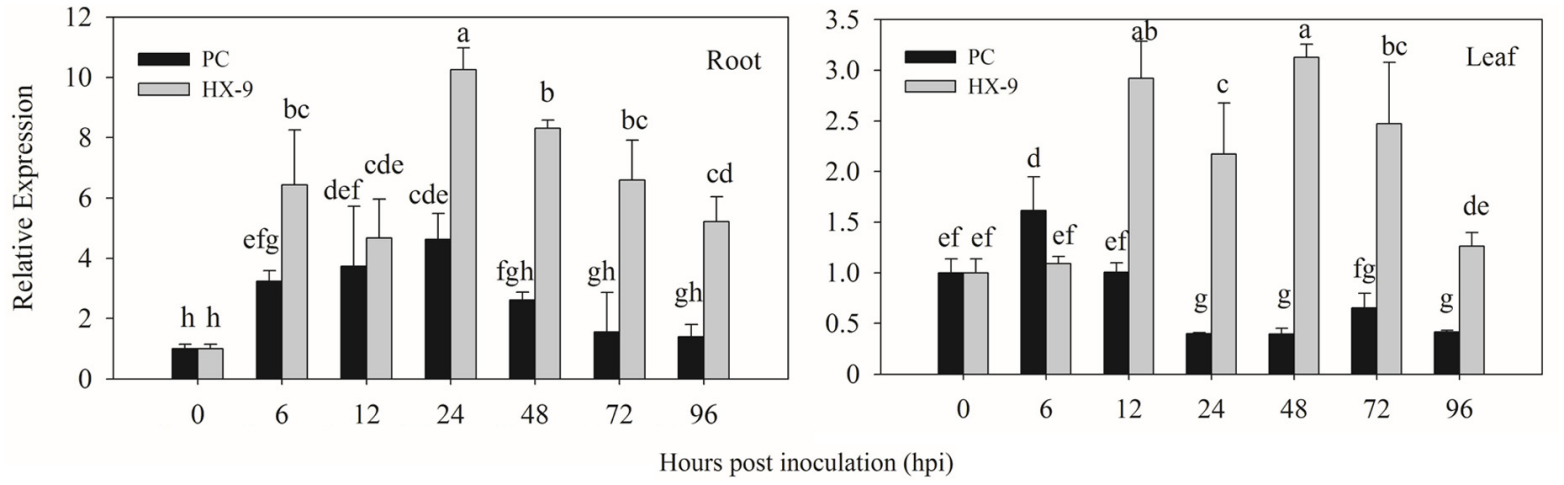
**Relative expression of *CaPTI1* in pepper plants exposed to *P. capsici* infection.** Relative expression of *CaPTI1* in the roots and leaves of pepper cultivar Y3 after inoculation with virulent (HX-9) or avirulent (PC) strains of *P. capsici*. Error bars presented means ± SD from three replicates. Bars with different lower case letters indicate significant differences using Duncan’s multiple range test (*p* < 0.05).

In virulent strain (HX-9) infection (**Figure 4**), the highest expression of *CaPTI1* in roots was detected at 24 h post inoculation (hpi) which was 10.24-fold of control, then decrease gradually and at 96 hpi the expression was 5.22-fold. The same trend of *CaPTI1* in roots was observed after inoculation with avirulent strain (PC), in which the expression of *CaPTI1* was increasing with the passage of time and reached to peak (4.62-fold) at 24 hpi and then decrease to the lowest (1.39-fold) at 96 hpi. Overall, the expression level of *CaPTI1* gene in the whole treatment with virulent strain was significantly higher than that with avirulent strain.

While in leaves, *CaPTI1* was up-regulated by virulent strain inoculation and the expression level was increased to 2.92-fold of control at 12 hpi, followed by a bit decrease, then reached to the peak (3.13-fold) at 48 hpi and at last decreased to 1.26-fold of control at 96 hpi. Less up regulation of *CaPTI1* was detected in the infection of avirulent strain except for a little increase at 6 hpi. Finally, the results showed that *CaPTI1* could be induced by *P. capsici*, especially in roots.

### Expression Analysis of *CaPTI1* Gene Under Abiotic Stresses

**FIGURE 5:**
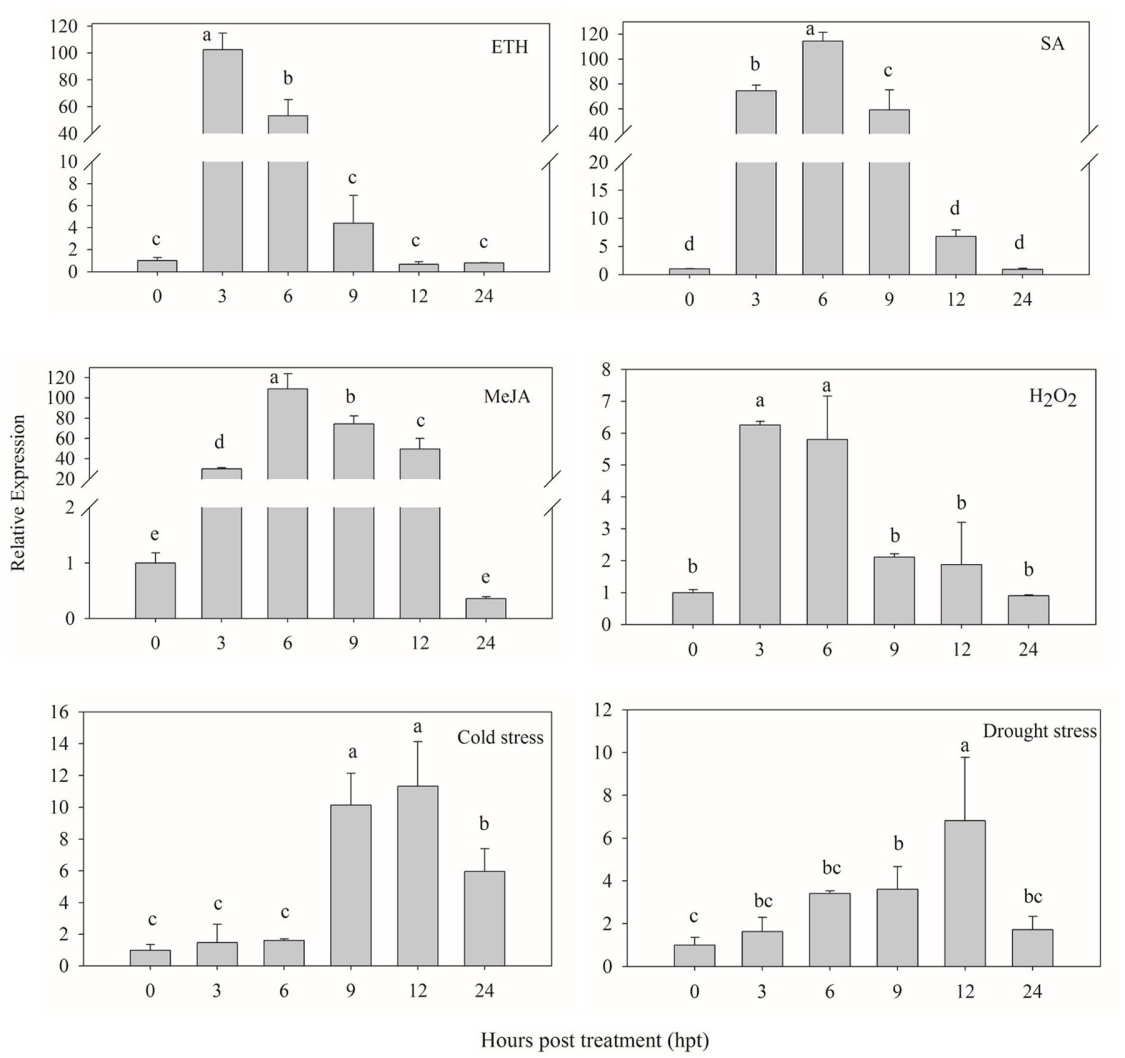
**Relative expression of *CaPTI1* responsed to various abiotic stresses.** Relative expression of *CaPTI1* gene in leaves of pepper plants after different treatments [Ethephon (ETH), salicylic acid (SA), Methyl Jasmonate (MeJA), hydogen peroxide (H_2_O_2_), cold stress and drought stress]. Error bars represent the mean ± SD of three independent biological replicates. Bars with different lower case letters indicate significant differences using Duncan’s multiple range test (*p* < 0.05).

The signaling molecules (ETH, SA, MeJA and H_2_O_2_) were used to investigate whether *CaPTI1* could be induced or not. As shown in **Figure 5**, *CaPTI1* in the leaves were strongly induced by foliar spraying of ETH, SA, and MeJA. As an *ERF*, *CaPTI1* in leaves showed a huge increase by ETH induction. The highest expression of *CaPTI1* was detected at 3 h post treatment (hpt), which was 102.57-fold of control. In the treatment of SA and MeJA, both the highest expression of *CaPTI1* was detected at 6 hpt, which were 114.53-fold and 108.80-fold of control respectively. While in the treatment with H_2_O_2,_ the significant up regulation of *CaPTI1* was observed at 3 and 6 hpt, which was 6.26-fold and 5.80-fold of control respectively.

Moreover, in the stresses of cold and drought, the expression of *CaPTI1* in leaves also showed a significant increase and rise to the peak at 12 hpt, which were 11.32-fold and 6.82-fold of control respectively.

### VIGS of *CaPTI1* Gene in Pepper Plants

To examine the effect of loss-of-function of the *CaPTI1* gene in pepper plants, VIGS was performed in pepper cultivar Y3. Empty vector (*pTRV2:00*) was used as a negative control. The *pTRV2:CaPDS* vector was used as a positive control, which will cause the silencing of *CaPDS* and induces a photo-bleaching phenotype in leaves.

**FIGURE 6:**
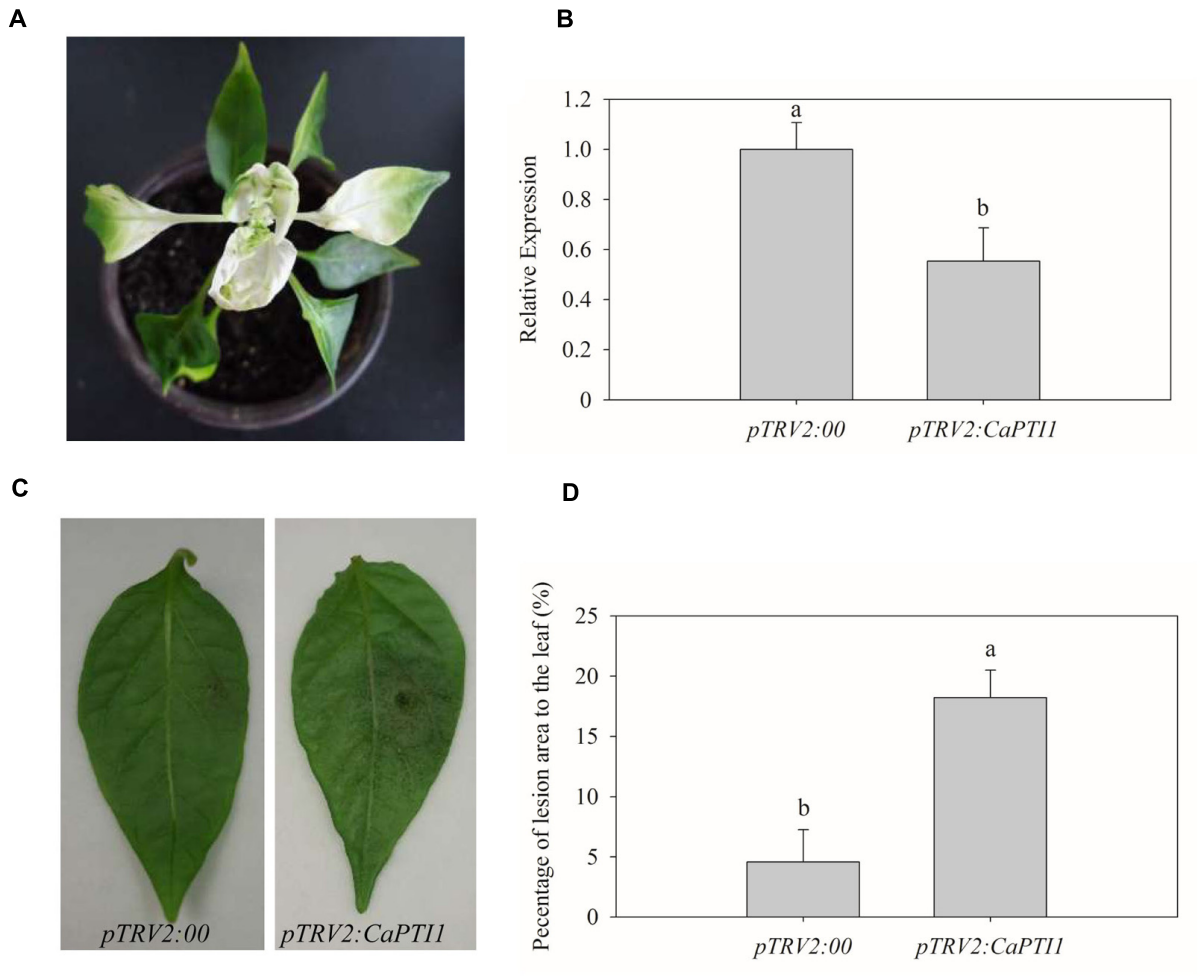
**Loss of function analysis of *CaPTI1* in leaves of pepper.** **(A)** The photo-bleaching phenotype in leaves of positive control pepper plants (*pTRV2:CaPDS*); **(B)** The relative expression of *CaPTI1* in leaves of *CaPTI1* silenced and negative control plants; **(C)** Disease symptoms developed on the detached leaves of silenced and control plants at 2 day post inoculation (dpi); **(D)** Percentage of the lesion area of the leaves inoculated with *P. capsici*. Error bars represent the mean ± SD of three independent biological replicates. Bars with different lower case letters indicate significant differences using Duncan’s multiple range test (*p* < 0.05).

Five weeks post inoculation, the phenotype of photo-bleaching was observed in leaves of positive control plants (**Figure 6A**). Then qRT-PCR analyses were performed to investigate the silencing efficiency of *CaPTI1* in leaves. *CaPTI1* in *CaPTI1* gene silenced plant exhibited half expression level of that in control (*pTRV2:00*) (**Figure 6B**). Then the third to fifth leaves from the top of control plants and *CaPTI1* silenced pepper plants (*pTRV2:CaPTI1*) were detached and inoculated by 20 μL zoospores suspension (10^5^ zoospores mL^-1^) of virulent strain of *P. capsici*. Disease symptoms were observed at 2 days post inoculation (dpi). The leaves of control plants exhibited less disease symptoms. While larger and more numerous lesions were observed in the leaves of *CaPTI1* silenced plants (**Figure 6C**). The quantitative analysis of the lesion area in the detached leaves showed a significant increase in *CaPTI1* silenced plants (**Figure 6D**).

**FIGURE 7:**
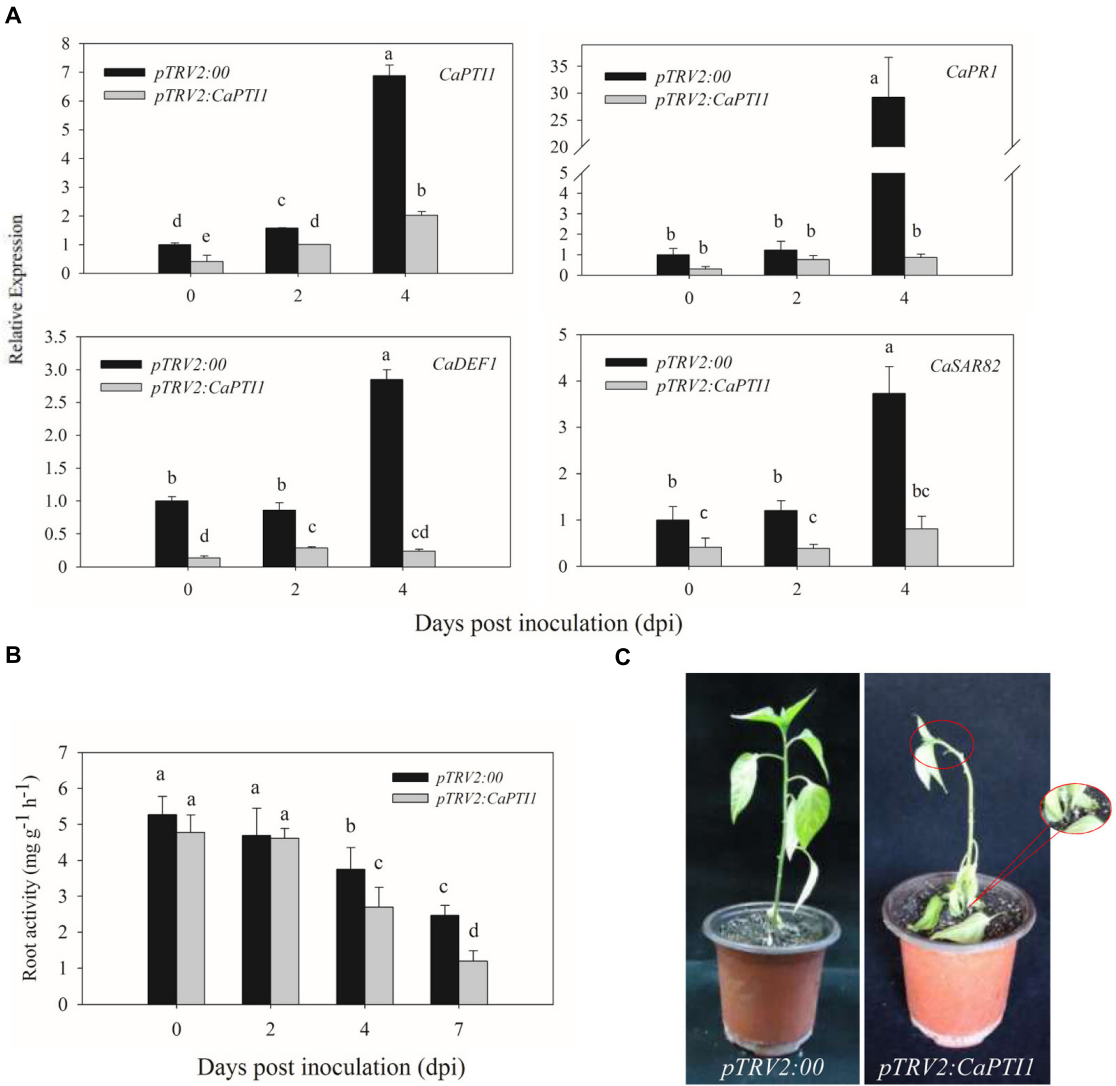
**The *CaPTI1* silenced pepper plants exhibit reduced resistance to *P. capsici*.** **(A)** The expression of *CaPTI1, CaPR1, CaDEF1*, and *CaSAR82* in the root of *CaPTI1* silenced and control plants were analyzed; **(B)** Root activity in *CaPTI1* silenced and control plants after inoculation with *P. capsici*. Values are the means ± SD from three independent experiments. Small letters represent significant differences (*p* < 0.05); **(C)** Disease symptoms of control and silenced plants were observed at 7 day post inoculation (dpi).

Besides, we also checked the expression of *CaPTI1* in roots of *CaPTI1* silenced plants and found it was only about 40% of the control (**Figure 7A**). After that we investigated whether silencing of *CaPTI1* altered the expression of defense related genes in roots during *P. capsici* infection. Significant decrease of *CaPR1* (a SA dependent gene) (Kim and Hwang, 2000), *CaDEF1* (a JA dependent gene) (Do et al., 2004) and *CaSAR82* (systemic acquired resistance gene) (Lee and Hwang, 2003) were detected in *CaPTI1* silenced plants. The time was expands to 4 dpi. Especially the *CaPR1* gene showed a really low expression (0.87-fold) in silenced plant, but a highest expression (29.23-fold) in control at 4 dpi. While at the same time, the expression of *CaPTI1* in silenced plant (2.02-fold) was still lower than that in control (6.88-fold).

In addition, the roots activity of silenced and control plants after inoculation with *P. capsici* were measured by TTC method. The *CaPTI1* silenced plants showed a lower root activity as compared to control, and a significant difference between silenced and control plants were detected at 4 dpi and enlarged to 7 dpi (**Figure 7B**). At 7 dpi disease symptoms of *Phytophthora* blight in pepper plants were observed. The *CaPTI1* silenced plants showed a serious disease symptom, such as the blacking and constricting of stem basement, the wilting and abscission of leaves and wilting of growing point of plant. While less and slightly disease symptoms were observed in the control plants (**Figure 7C**).

## Discussion

The AP2/ERF family is one of the largest transcription factor family and demonstrated to be involved in the regulation of plant development and response to biotic and abiotic stresses (Gutterson and Reuber, 2004; Wessler, 2005; Mizoi et al., 2012). In this study, we identified a new ERF in pepper, which named *CaPTI1* and belonged to the ERF family.

Sakuma et al. (2002) divided the ERF family into ERF subfamily and DREB subfamily according to the amino acids difference at position 14th and 19th in AP2/ERF domain. In addition, several ERFs which contain alanine and aspartic acid at 14th and 19th position of the AP2/ERF domains could bind to both the DRE and GCC boxes (Park et al., 2001; Wang et al., 2004). The 14th alanine and the 19th aspartic acid in the AP2/ERF domain of CaPTI1 suggested a member of ERF subfamily. And sequence character analysis showed that CaPTI1 does not contain an EAR motif, which maybe indicate the activation role of CaPTI1 in plant defense response.

*Phytophthora capsici* mainly invades the pepper plant by roots and *CaPTI1* has a highest expression in roots before inoculation. After infection of *P. capsici* the expression level of *CaPTI1* in roots got a significant increase. These possibly indicated that *CaPTI1* is involved in the interaction of pepper and *P. capsici. CaPTI1* could not only be induced by *P. capsici*, but also strongly induced by ETH, SA and MeJA. These hormones as signaling molecules had been showed to play crucial roles in defense response pathway (Dong, 1998; Reymond and Farmer, 1998; Pieterse and van Loon, 1999). The SA signaling transduction pathway usually acts as antagonism with ET/JA pathway. *ERF1* of *Arabidopsis* was found to be a key element in the integration of both ET and JA pathways for the regulation of defense response genes (Lorenzo et al., 2003). Besides, *AtERFs* may be involved in the cross-talk between SA and JA signaling pathways and important for plant defense to pathogen attack (Onate-Sanchez and Singh, 2002). *ERF* transcription factor *ORA59* was supposed to be the integrator of JA and ET signaling pathways (Zarei et al., 2011). The *ERF* transcription factor in tomato, *Pti4* could be induced by SA, JA, and ETH. While *Pti5* and *Pti6* could not be induced by SA in tomato (Gu et al., 2000). Further studies found that *Pti4*, *Pti5*, and *Pti6* may be implicated indirectly in regulating the SA response through interaction with other *TFs*, and all of them could activate the expression of *PR* genes and play important roles in plant defense (Gu et al., 2002; Chakravarthy et al., 2003). All of these indicated that *CaPTI1* may be involved in the pathways which were mediated by the signaling molecules SA, MeJA and ETH.

We also detected the up regulation of *CaPTI1* by the spraying of H_2_O_2_. Reactive oxygen species (ROS) are produced constantly during normal plant growth and development and also perform essential roles as highly specific signaling molecules under stress conditions (Apel and Hirt, 2004). Plant defense hormones SA and JA could also modulate the plants ROS status (Mittler et al., 2011). *ERF6* was turned out to be a regulator of ROS in *Arabidopsis* and response to biotic and abiotic stresses (Sewelam et al., 2013). Moreover, *CaPTI1* could be induced by the cold and drought stresses which indicated *CaPTI1* response to abiotic stresses. Over-expression of the tobacco *Tsi1* gene also enhances resistance against pathogen attack and osmotic stress in tobacco (Park et al., 2001). *StERFs* also were shown to be regulated by drought stress (Wang et al., 2015). All these suggested the multiple role of *CaPTI1* in pepper plants response to diverse stresses.

We silenced the *CaPTI1* gene in leaves and roots of pepper plants successfully by VIGS. Detached leaves assay were frequently used to investigate the plant disease resistance to the invasion of pathogens (Wang et al., 2013c; Dong et al., 2015; Tian et al., 2015). After inoculation of *P. infestans* larger disease lesions were observed on the leaves of *StERF3* over-expressed potato lines as compared to the wild type, which indicated the repressor role of *StERF3* in defense response to *P. infestans* in potato. Less disease lesion were observed on the leaves of *GmERF5* transgenic soybean lines after infection with *P. sojae* (Dong et al., 2015). Detached leaves assay with the inoculation of *P. capsici* were also performed in this study after the silencing of *CaPTI1* in pepper, and then larger lesion area was observed in leaves of *CaPTI1* silenced plants as compared to control. This indicated that silencing of *CaPTI1* in pepper leaves make it more susceptible to *P. capsici* infection.

In addition, the roots of pepper were focused on because *Phytophthora* blight is a soil borne disease. Significant decrease of defense related genes *CaPR1*, *CaDEF1*, and *CaSAR82* was detected in roots of *CaPTI1* silenced plants when compared to the control plants, especially for the *CaPR1*, which is a SA dependent gene (Kim and Hwang, 2000). As mentioned before, *ERFs* could specially recognize and bind to the GCC-box. *Pti4* could regulate defense related genes expression via both GCC-box and non-GCC-box (Chakravarthy et al., 2003). Two GCC-box were found in the upstream promoter region (about 1500 bp) of *CaPR1* according the database of pepper genome (Kim et al., 2014). The silencing of *CaPTI1* maybe decrease the expression of *CaPR1* by affecting the binding of GCC-box which is in the promoter region of *CaPR1*. Moreover, Kim and Hwang (2000) found that treatment with SA in combination with ETH, caused a lower expression of the *CaPR1* in pepper leaves than the treatment with ETH alone, and pepper stems will synthesize ethylene at an exponential rate soon after infection with either virulent or avirulent strains of *P. capsici*. As *Pti4, Pti5*, and *Pti6* were supposed to be involved in the regulation of ethylene production in the plant singly or in combination (Gu et al., 2000). The silencing of *CaPTI1* may reduce the ethylene production of pepper plants. As a result, the silencing of *CaPTI1* may enhance the antagonism between SA and ET pathways by changing the ratio of SA to ET in *CaPTI1* silenced pepper plants and then decrease the expression of *CaPR1*. The enhanced antagonism may also affect the expression of *CaPTI1* in turn.

*CaPTI1* silenced pepper plants showed a significant decrease in the expression of *CaDEF1* (a JA-dependent gene) compare to the control. JA and ETH usually appear to function synergistically to induce *PDF1.2* (a JA-dependent gene) in *Arabidopsis*, and *osmotin* and *PR1b* in tobacco (Xu et al., 1994; Penninckx et al., 1998; Norman-Setterblad et al., 2000). *CaDEF1* may act as the downstream of JA/ET pathway and be regulated by *CaPTI1* indirectly. The silencing of *CaPTI1* maybe also decreases the synergism between JA and ET by decreasing the ethylene production in pepper plants. *CaSAR82* serves as a molecular marker for the onset of ISR in pepper plants and could not only be induced by pathogens (*Xanthomonas campestris* pv. *vesicatoria*, *Colletotrichum coccodes*, and *Phytophthora capsici)*, but also the signaling molecule (SA, JA, ETH and H_2_O_2_) and various stresses (Lee and Hwang, 2003). ISR was generally regulated by JA and ET. The significant decrease of *CaSAR82* in *CaPTI1* silenced pepper plants maybe indicated the systemic resistance mediated by JA/ET was weakened by the silencing of *CaPTI1*.

Besides a significant decrease of root activity was observed at 4 dpi in *CaPTI1* silenced plants as compared to control. The root activity which was measured by the TTC method was used as a cell vitality indicator (Chen et al., 2006). The cell vitality of root indicated the injury degree of pepper roots after the infection of *P. capsici.* Yeom et al. (2011) used this assay to measure TTC reductase activity in the roots of pepper cultivars CM334 (resistant to *P. capsici*) and Chilsungcho (susceptible to *P. capsici*) after inoculation with *P. capsici*. They found that significant differences were observed from 24 to 48 hpi, showing two or three times more activity in CM334 than in Chilsungcho. Moreover Wang et al. (2013b) also found that there is a significant difference in pepper plants after inoculation with the incompatible and compatible strains of *P. capsici*, and the root activity was much higher in the infection of incompatible strain than that of compatible strain. In this study, a significant decrease of root activity was observed at 4 dpi in *CaPTI1* silenced plants when compared to the control plants. This may indicate the serious injury to the roots of *CaPTI1* silenced pepper plants by *P. capsici*. Both of the significant decreased root activity and the reduced expression of defense related genes indicated the weakness of defense response in *CaPTI1* silenced pepper plants and suggested the significant role of *CaPTI1* in defense response to the infection of *P. capsici*.

Taken together, *CaPTI1* contains an AP2/ERF domain and belongs to the ERF family. *CaPTI1* could not only responses to the infection of *P. capsici* and the stresses of cold and drought, but also be induced by SA, MeJA, ETH and H_2_O_2_. VIGS of *CaPTI1* in pepper could weaken the defense response significantly by reducing the expression of defense related genes *CaPR1*, *CaDEF1* and *CaSAR82* and also the root activity. However, more detailed researches are still needed to reveal the exact role of *CaPTI1* in the regulation of defense response to *P. capsici* in pepper.

## Author Contributions

J-HJ, D-WL, and Z-HG conceived the research. J-HJ, H-XZ, J-YT, and M-JY performed the research. J-HJ and H-XZ performed statistical analyses. J-HJ wrote the paper. J-HJ, AK and Z-HG revised the paper. D-WL and Z-HG provided the materials and resources for the research. J-HJ, H-XZ, and Z-HG performed the integrity of the work. All authors read and approved the final manuscript.

## Conflict of Interest Statement

The authors declare that the research was conducted in the absence of any commercial or financial relationships that could be construed as a potential conflict of interest.

## Footnotes

1 http://www.expasy.ch/tools/pi_tool.html

2 http://www.ncbi.nlm.nih.gov/Structure/cdd/wrpsb.cgi
