# Supplementary material for: A New Ethylene-Responsive Factor CaPTI1 Gene of Pepper (Capsicum annuum L.) Involved in the Regulation of Defense Response to Phytophthora capsici
Source: Front Plant Sci. 2016 Jan 8;6:1217. doi: 10.3389/fpls.2015.01217 (PMC4705296; doi:10.3389/fpls.2015.01217)
Supplement: Supplementary file 1 [file Table_1.DOC]

**Table S1.** Primers used in the study

| Primers | GenBank NO. | sequence (5'-3') |
| --- | --- | --- |
| *CaPTI1* | KJ690096 | F: TCTGTAATTAGTTATGGTTCCAACTC  R: GAAAAAATAGAAAATAGAATGTG |
| *CaUbi*3 | AY486137 | F: TGTCCATCTGCTCTCTGTTG  R: CACCCCAAGCACAATAAGAC |
| qRT*_CaPTI1_* |  | F: GGTTCCAACTCACCAAAGTGATC  R: TTCTTGCCTATGGTTTGTGATGG |
| VIGS*_CaPTI1_* |  | F: CGGAATTCTTTCTCAAGACGCTAAGACA  R: CGGGATCCGCTAATTATCCTAAGCAATCTTT |
| *CaPR1* | AF053343 | F: TGGAGACTGCAGGATGCAACACT  R: TACCACCCATTGTTGCACCGAAC |
| *CaDEF1* | AF442388 | F: CAAGGGAGTATGTGCTAGTGAGAC  R: TGCACAGCACTATCATTGCATAC |
| *CaSAR82* | AF112868 | F: CAGGGAGATGAATTCTGAGGC  R: CATATGAACCTCTATGGATTTCTG |

F: forward primer; R: reverse primer.
